# Thymol improves the growth performance of blue foxes by regulating the gut microbiota

**DOI:** 10.3389/fmicb.2024.1368293

**Published:** 2024-06-14

**Authors:** Chongshan Yuan, Siqi Chen, Rui Sun, Lili Ren, Tiancheng Zhao, Min Wu, Aiwu Zhang

**Affiliations:** College of Animal Science and Technology, Jilin Agricultural University, Changchun, China

**Keywords:** thymol, blue fox, growth performance, serum biochemical biochemistry, intestinal morphology, gut microbiota

## Abstract

**Introduction:**

The drawbacks of using antibiotics as feed additives for blue foxes have gradually become apparent; moreover, thymol has wide-spectrum antimicrobial activity and has the potential to replace antibiotics in various animals. However, there are few reports on the effects of thymol on blue foxes.

**Methods:**

This study aimed to investigate the effects of different concentrations of thymol on the growth performance, apparent nutrient digestibility, serum biochemical indicators, intestinal morphology, and gut microbiota of blue foxes. Twenty-four male blue foxes (120 ± 5 d) of similar weight (6.05 ± 0.16 kg) were randomly divided into 4 groups. 0, 100, 200, and 300 mg/kg thymol were added to the basal diets of groups C, L, M, and H, respectively.

**Results:**

Compared with those in the C group, the addition of 100 mg/kg thymol to the diet significantly increased organic matter (OM) digestibility, crude protein (CP) digestibility, immunoglobulin (Ig) A, IgM, the VH of the duodenum, the CD of the jejunum, the VH of the ileum, and the VH/CD of the ileum (*P* < 0.05) and strongly significantly increased IgG (*P* < 0.01). The addition of 200 mg/kg thymol to the diet increased the VH/CD of the duodenum (*P* < 0.05). The addition of 300 mg/kg thymol to the diet significantly increased the VH and CD of the jejunum (*P* < 0.05). The addition of 200 mg/kg and 300 mg/kg thymol to the diets increased the final weight (FW) (*P* < 0.05). Adding 100 mg/kg thymol significantly increased the levels of interleukin-4 (IL-4) and catalase (CAT) compared with those in the other groups (*P* < 0.05). 16S rRNA gene detection revealed that thymol can change the abundances of *Bifidobacterium*, *Fusobacterium*, *Allobaculum*, *Streptococcus*, *Megasphaera*, and *Lactobacillus* in the gut.

**Conclusion:**

The addition of thymol to diets can increase the abundance of *Bifidobacterium*, *Fusobacterium*, and *Allobaculum*, which may contribute to improving the growth performance of blue foxes.

## 1 Introduction

Fox pelt, mink pelt, and Persian lamb pelt are the three major types of fur in the world, among which blue fox skin is an important raw material for making high-end fur clothing and accessories, known as the “soft gold” in fur (Ylinen et al., 2020). Blue foxes are widely bred in northern China, and their breeding scale is constantly expanding. However, the drawbacks of using antibiotics as feed additives for blue foxes, such as increased resistance to pathogenic microorganisms, inhibition of blue fox growth performance, and the generation of drug residues, have gradually become prominent (Bhardwaj et al., 2022). Therefore, finding alternative products for antibiotics has become an important direction for the development of sustainable animal husbandry. Thymol is a safe and green feed additive that can enhance the immune system of animals to resist the invasion of exogenous pathogens and has potential value as a substitute for antibiotics (Marchese et al., 2016). Thymol, as a potential feed additive to replace antibiotics, has been applied in various animal feed sources. A previous study revealed that the addition of 1% thymol to the diet of weaned piglets can increase the serum concentrations of immunoglobulin (Ig) A and IgM and alleviate the harmful effects of *Salmonella typhimurium* on piglet growth performance (Trevisi et al., 2007). It has been reported that thymol can reduce the inflammatory response caused by *Staphylococcus aureus* infection by regulating extracellular vesicles (Kwon et al., 2019). Furthermore, thymol enhances barrier function and reduces the production of reactive oxygen species and the expression of proinflammatory cytokine genes in epithelial cells (Omonijo et al., 2019). Similarly, Liang et al. (2014) reported that thymol may inhibit the production of tumor necrosis factor-α (TNF-α) and interleukin-β (IL-β) in lipopolysaccharide (LPS)-stimulated mouse mammary epithelial cells. In addition, thymol can accelerate the metabolism and renewal rate of intestinal villous epithelial cells, reducing the risk of pathogen invasion (Ghiselli et al., 2022). Thymol may improve animal production performance by regulating the gut microbiota. Thymol can improve animal production performance by regulating the gut microbiota. Betancourt et al. (2014) showed that adding thymol to the basic diet of male chicks improved the microbial community structure and reduced the risk of disease and mortality. Adding 30 mg/kg thymol to the basic diet of male turkeys can increase the number of *Lactobacillus* strains in the cecum, reduce the number of *Escherichia coli*, and promote the growth and development of turkeys (Giannenas et al., 2014). Dietary thymol supplementation can promote the digestion and absorption of nutrients in piglets, reduce diarrhea, have a positive impact on the development of intestinal morphology, and improve the gut microbiota (Diao et al., 2015). The addition of 100 mg/kg thymol to the sow diet can increase the number of *Lactobacillus*, *Escherichia coli*, and *Enterococcus* in feces and increase the average daily weight gain (ADG) of suckling piglets (Tan et al., 2015). Thus, thymol has the potential to replace antibiotics, but its application in fur animal growth performance, especially in blue foxes, has not yet been reported. Therefore, the purpose of this study was to investigate the effects of thymol on the growth performance, apparent nutrient digestibility, serum indices, intestinal morphology, and gut microbiota of blue foxes and to provide a theoretical and practical foundation for the application of thymol in blue foxes.

## 2 Materials and methods

### 2.1 Experimental animals

The experimental animals were purchased from the Beidahuang Blue Fox Breeding Farm in Jilin Province, China. Twenty-four healthy male blue foxes aged 120 ± 5 days and weighing 6.05 ± 0.16 kg were selected. The blue foxes were randomly divided into 4 groups, with 6 in each group. Each blue fox was kept in an individual cage (1.0 m × 1.0 m). The C group was fed basic feed (Table 1), while the L, M, and H groups were supplemented with 100, 200, and 300 mg/kg thymol in the basic diet, respectively. Feed was provided at 06:30 and 16:00, and water was provided *ad libitum*. The experiment was conducted at the teaching experimental base of Jilin Agricultural University, and the test period consisted of a 5-day acclimation phase and a 30-day feeding trial. The blue foxes were vaccinated with basic vaccines (rabies vaccine and canine parvovirus vaccine) before the experiment.

**TABLE 1:** Composition and nutrient levels of the basal diet (dry matter basis).

Ingredients	Content (%)	Nutrient levels	Content (g/kg)
Extruded corn	36.00	ME/(MJ/kg)	14.32
Soybean meal	10.00	CP	25.88
Corn gluten	6.50	EE	19.65
Distillers grain	3.50	Methionine	0.79
Corn germ	3.10	Cysteine	0.31
Meat meal	8.00	Lysine	0.60
Fish meal	11.00	Ca	1.86
Blood meal	2.00	P	1.31
Feather meal	2.00	Cu/(mg/kg)	3.84
Soybean oil	15.00		
Ca(H_2_PO4)_2_	2.25		
NaCl	0.20		
Premix	0.45		

ME, energy; CP, crude protein; EE, ether extract. The premix provided the following per kg of diet: vitamin A1 000 000 IU, vitamin D3 2 000 000 IU, vitamin E 6 000 IU, vitamin B1 500 mg, vitamin B2 700 mg, vitamin K3 100 mg, vitamin C 40 000 mg, nicotinic acid 4 000 mg, pantothenic acid 70 mg, choline 30 000 mg, Fe 8 200 mg, Mn 1 200 mg, Zn 5 200 mg, I 50 mg, Se 20 mg, and Co 50 mg. ME was a calculated value, while the others were measured values.

### 2.2 Sample collection

Fecal and feed samples were collected and stored at −20°C daily, and daily food intake and fecal weight were recorded during the last 5 days of the feeding trial. For nitrogen analysis, daily samples of 3% total feces were collected and kept in wide-mouth vials containing 20 mL of 10% H_2_SO_4_. On the last day of the feeding trial, 8 mL of blood was collected from the hind leg vein and placed in a procoagulant collection vessel. After centrifugation at 4,000 rpm for 10 min, the serum was separated and divided into 1.5 mL centrifuge tubes, which were stored at −80°C. At the end of the trial, three blue foxes were randomly selected from each group and humanely slaughtered via electric shock according to the guidelines outlined in the Welfare of Animals Kept for Fur Production. The contents of the rectum were placed in 2 mL sterilized centrifuge tubes and then stored at −80°C. The duodenum, jejunum, and ileum samples were washed clean with physiological saline and soaked in 10% formaldehyde solution for at least 24 h for hematoxylin and eosin (H&E) staining.

### 2.3 Growth performance and apparent nutrient digestibility

On the first and last days of the feeding trial, the weight of each blue fox was measured in the morning without feeding, the initial weight (IW) and final weight (FW) were recorded, and the ratio of consumed feed (F/G) to ADG was calculated. At the end of the trial, the fecal and feed samples were dried in a forced-air oven at 65°C for 72 h. The samples were crushed in a fodder grinder, and samples smaller than 1 mm were filtered out. To determine the nutritional content of the fecal and feed samples, routine chemical tests were performed following the association of official analytical chemists (AOAC) guidelines (Feldsine et al., 2002). Briefly, the organic matter (OM) content was calculated by measuring the ash content, which was obtained by burning the samples at 550°C for 8 h. The N content was determined using a LECO FP-528N/Protein Tester (LECO, Corporation), and the crude protein (CP) content in the sample was calculated by the following equation: CP = N × 6.25. Ether extract (EE) was extracted from the samples using the diethyl ether extraction–submersion method, and their contents were calculated. The apparent nutrient digestibility was calculated using the following formula: apparent nutrient digestibility (%) = (nutrient intake-nutrient excreta) / nutrient intake × 100.

### 2.4 Serum immune and antioxidant indices

Immunoglobulin A (IgA), immunoglobulin G (IgG), immunoglobulin M (IgM), interleukin-4 (IL-4), superoxide dismutase (SOD), catalase (CAT), and blood urea nitrogen (BUN) were measured using commercial kits (Nanjing Jiancheng Bioengineering Research Institute, Nanjing, China) according to the manufacturer’s guidelines. Briefly, 10 μL of sample and 40 μL of Sample Diluent were added to the testing well first. Then, 100 μL of HRP-Conjugate Reagent was added to each well, and the plates were incubated for 60 min at 37°C. Each well was washed 5 times with Wash Solution. Then, Solution A, Solution B, and Stop Solution were added to each well. Finally, the results were measured at 450 nm within 15 min.

### 2.5 Hematoxylin and eosin (H&E) staining and intestinal morphology

The 0.5–1 cm samples of the duodenum, jejunum, and ileum were placed into embedding boxes and rinsed with tap water for 12 h. Afterward, the embedding boxes were immersed overnight in 50% ethanol solution, followed by soaking them in 75, 85, 90, 95, and 100% ethanol for 1 h each and soaking them in xylene I and xylene II for 30 min each. Finally, the sections were paraffin embedded and cut to a thickness of approximately 4.5 μm. The sections were dewaxed in xylene for 7 min and then placed in 95, 85, 70, and 50% alcohol for 2 min each. Then, the sections were soaked in hematoxylin staining solution for 10 min, 1% hydrochloric acid solution for 30 s, water for 30 s, 50, 70, 80, and 90% ethanol solution for 2 min. The sections were subsequently stained with eosin staining solution for 10–20 s. The sections were placed in 95% ethanol, anhydrous ethanol, and xylene twice for 10 min each. Finally, the sections were sealed with neutral resin and observed under an optical microscope (Olympus, Tokyo, Japan). The intestinal morphology was determined under 40× magnification, 5 positions on each section were randomly selected, the villus height (VH) and crypt depth (CD) were measured using Image-Pro Plus 6.0 software, and the VH/CD ratio was calculated.

### 2.6 Gut microbiota

16S rRNA V3-V4 sequencing was used to detect the rectum content. The DNA in the rectum was extracted using a Qiagen magnetic bead extraction kit (Qiagen, Valencia, California, USA) according to the manufacturer’s instructions. Agarose gel electrophoresis was used to evaluate the purity and concentration of the DNA. The sample DNA was diluted to 1 ng/μL with sterile water as a template for PCR amplification, and the sequencing primers used were F (ACTCCTACGGGGGGAGAGCA) and R (GGACACHVGGGTWTCTAAT). The PCR products were identified and purified by 2% agarose gel electrophoresis, and the target band was cut and recovered (Thermo Scientific Company GeneJET adhesive recovery kit). Thermofisher Ion Plus Fragment Library Kit 48 (RXNS) was used to construct the library. Then, sequencing was performed on the Illumina NovaSeq 6000 platform to obtain end reads, which were merged into the original labels using FLASH version 1.2.7. To obtain the final reliable data (high-quality clean tags), Uparse (version 7.0.10012) was used to eliminate the chimeric sequence. The tags were compared to the SILVA database (version 138), and the DADA2 plugin was then used to filter the sequences for quality, denoise them, combine them, and eliminate chimeras (Callahan et al., 2016). The mothur technique and SSUrRNA database of SILVA132 (threshold set to 0.8) were utilized to perform species annotation on operational taxonomic unit (OTU) sequences. Bioinformatics analysis of the microbiome was performed using QIME2 2019, and the α and β diversity of the samples was calculated (Lozupone and Knight, 2005). The abundances of different bacteria were quantified with qPCR (StepOne™ Real-Time PCR System, Applied Biosystems) according to a previous study (Alberoni et al., 2021). The screening value of the linear discrimination criterion (LDA score) was 2 according to the LEfSe program for intergroup differential species analysis. Spearman analyzed the correlation between growth performance and the gut microbiota.

### 2.7 Statistical analysis

All the graphs were generated using GraphPad Prism version 8 and PowerPoint version 2021. The statistical analysis of the data was performed using the general linear model program SPSS (22.0, IBM Co. Limited, Chicago, USA). Duncan multiple comparisons in one-way analysis of variance (ANOVA) were used to test the significance of differences between groups. The data were averaged to one value per animal, and the data were presented as the mean and standard deviation (mean ± SD). *P* < 0.05 was considered statistically significant, *P* < 0.01 was considered extremely significant, and *P* > 0.05 was considered not statistically significant. STAMP software (T test) was used to analyze the differences in microbiota abundance between groups, and the Benjamini-Hochberg FDR multiple test correction method was used to control the false positive rate.

## 3 Results

### 3.1 Effect of thymol on the growth performance of blue foxes

As shown in Table 2, the addition of 200 mg/kg and 300 mg/kg thymol to the basic diet significantly increased the FW compared with that in the C group (*P* < 0.05). The IW, ADG, and F/G ratio did not significantly differ among the groups (*P* > 0.05).

**TABLE 2:** The effect of thymol on the body weight of blue foxes.

Parameter	C (0 mg/kg)	L (100 mg/kg)	M (200 mg/kg)	H (300 mg/kg)
IW/kg	6.02 ± 0.18	6.00 ± 0.12	6.02 ± 0.18	6.18 ± 0.08
FW/kg	7.78 ± 0.36^b^	8.08 ± 0.18^ab^	8.14 ± 0.18^a^	8.15 ± 0.24^a^
ADG/g	54.28 ± 15.00	58.86 ± 6.91	58.58 ± 5.84	58.18 ± 9.35
F/G	4.41 ± 0.81	4.30 ± 0.51	3.90 ± 0.28	3.86 ± 0.50

IW, initial weight; FW, final weight; ADG, average daily weight gain; F/G, ratio of consumed feed to weight gain. 0 mg/kg (C group), 100 mg/kg (L group), 200 mg/kg (M group), and 300 mg/kg (H group) thymol were added to the basic diets of the blue foxes. The values are expressed as the means ± SD. Means for different lowercase letters are significantly different (*P* < 0.05).

### 3.2 Effect of thymol on the apparent digestibility of nutrients in blue foxes

The apparent nutrient digestibility of blue foxes is shown in Table 3. Supplementing basic diets with 100 mg/kg thymol increased OM digestibility compared with that in the C group (*P* < 0.05). Moreover, compared with that of the C group, the digestibility of CP in the L group increased significantly (*P* < 0.05). However, there were no significant differences in EE digestibility among the groups (*P* > 0.05).

**TABLE 3:** Effect of thymol on the apparent nutrient digestibility of blue foxes (%).

Parameter	C (0 mg/kg)	L (100 mg/kg)	M (200 mg/kg)	H (300 mg/kg)
OM	82.04 ± 6.12^b^	88.73 ± 1.48^a^	84.59 ± 3.12^ab^	84.99 ± 2.20^ab^
EE	93.85 ± 3.15	94.70 ± 1.54	94.00 ± 1.50	93.90 ± 2.21
CP	79.37 ± 0.59^b^	85.84 ± 1.89^a^	84.38 ± 0.81^ab^	80.78 ± 5.26^ab^

OM, organic matter digestibility; EE, ether extract digestibility; CP, crude protein digestibility. 0 mg/kg (C group), 100 mg/kg (L group), 200 mg/kg (M group), and 300 mg/kg (H group) thymol were added to the basic diets of the blue foxes. The values are expressed as the means ± SD. Means for different lowercase letters are significantly different (*P* < 0.05).

### 3.3 Effect of thymol on the serum biochemical indicators of blue foxes

As shown in Table 4, the content of IgG in the L group was significantly greater than that in the C group (*P* < 0.01). Moreover, the addition of 100 mg/kg thymol to the basic diet increased the contents of IgA and IgM compared with those in the C group (*P* < 0.05). The contents of IL-4 and CAT in the L group were greater than those in the other groups (*P* < 0.05). The serum IgM content in the L group was significantly greater than that in the H group (*P* < 0.05). However, there were no significant differences in the levels of SOD or BUN among the groups (*P* > 0.05).

**TABLE 4:** Effect of thymol on the serum biochemical indicators of blue foxes.

Parameter	C (0 mg/kg)	L (100 mg/kg)	M (200 mg/kg)	H (300 mg/kg)
IgG (μg/mL)	3.38 ± 0.16^B^	4.44 ± 0.10^A^	4.04 ± 0.36^AB^	3.85 ± 0.27^AB^
IgA (μg/mL)	432.85 ± 7.62^B^	498.30 ± 36.07^A^	483.45 ± 11.39^ab^	466.48 ± 23.89^ab^
IgM (μg/mL)	112.96 ± 25.68^c^	361.85 ± 33.82^A^	338.15 ± 8.47^ab^	301.11 ± 44.07^B^
IL-4 (pg/mL)	11.94 ± 0.74^B^	14.38 ± 1.17^A^	11.84 ± 0.36^B^	11.89 ± 0.70^B^
SOD (U/mL)	4.05 ± 4.33	3.89 ± 0.67	3.44 ± 0.03	3.70 ± 0.23
CAT (U/mL)	51.42 ± 0.40^B^	55.01 ± 2.42^A^	50.95 ± 1.02^B^	51.14 ± 0.84^B^
BUN (mmol/L)	9.09 ± 0.56	8.07 ± 0.19	8.84 ± 0.32	9.82 ± 0.36

IgG, immunoglobulin G; IgA, immunoglobulin A; IgM, immunoglobulin M; IL-4, interleukin-4; SOD, superoxide dismutase; CAT, catalase; BUN, blood urea nitrogen. 0 mg/kg (C group), 100 mg/kg (L group), 200 mg/kg (M group), and 300 mg/kg (H group) thymol were added to the basic diets of the blue foxes. The values are expressed as the means ± SD. Means for different lowercase letters are significantly different (*P* < 0.05). Means for different capital letters are significantly different (*P* < 0.01).

### 3.4 Effect of thymol on the intestinal morphology of blue foxes

**FIGURE 1:**
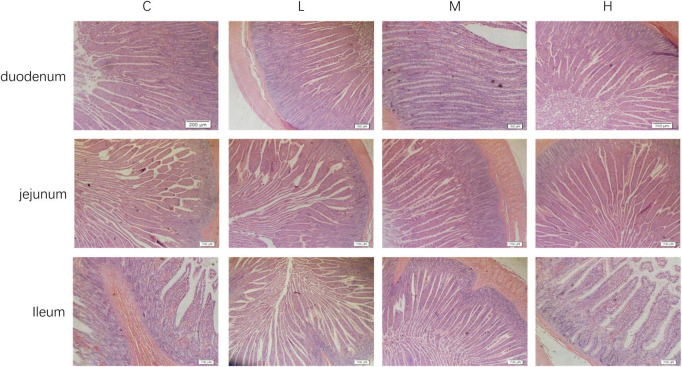
Intestinal morphology of blue foxes. 0 mg/kg (C group), 100 mg/kg (L group), 200 mg/kg (M group), and 300 mg/kg (H group) thymol were added to the basic diets of the blue foxes.

The intestinal morphologies of the duodenum, jejunum, and ileum are shown in Figure 1. From Table 5, it can be seen that compared with the Group C, adding 100 mg/kg thymol to the basic diet significantly increased the VH of the duodenum (*P* < 0.05). Adding 200 mg/kg thymol to the basic diet significantly increased the VH/CD of the duodenum (*P* < 0.05). Compared with the C group, the basic diet supplemented with 300 mg/kg thymol significantly increased the VH of the jejunum (*P* < 0.05). Supplementing basic diets with 100 or 300 mg/kg thymol significantly increased the CD of the jejunum (*P* < 0.05). The VH and VH/CD of the ileum in the L group were significantly greater than those in the C group (*P* < 0.05). The CD of the duodenum, VH/CD of the jejunum, and CD of the ileum were not significantly different among the groups (*P* > 0.05).

**TABLE 5:** Effect of thymol on the intestinal morphology of blue foxes.

Parameter	C (0 mg/kg)	L (100 mg/kg)	M (200 mg/kg)	H (300 mg/kg)
**Duodenum**				
VH/μm	801.94 ± 86.44^b^	1,302.72 ± 303.04^a^	1,231.24 ± 287.85^ab^	1,199.86 ± 250.67^ab^
CD/μm	351.34 ± 55.34	440.50 ± 64.09	379.99 ± 62.69	354.02 ± 45.31
VH/CD	2.29 ± 0.11^b^	2.93 ± 0.27^ab^	3.23 ± 0.32^a^	2.92 ± 0.21^ab^
**Jejunal**				
VH/μm	916.19 ± 97.26^b^	1,170.64 ± 174.32^ab^	1,039.64 ± 99.83^ab^	1,274.16 ± 93.06^a^
CD/μm	297.65 ± 34.21^b^	416.35 ± 66.35^a^	393.96 ± 57.19^ab^	407.12 ± 32.50^a^
VH/CD	2.77 ± 0.11	2.81 ± 0.03	3.07 ± 0.22	3.16 ± 0.50
**Ileum**				
VH/μm	772.87 ± 49.01^b^	1,049.34 ± 134.69^a^	900.90 ± 77.93^ab^	947.03 ± 108.69^ab^
CD/μm	254.88 ± 28.18	237.13 ± 17.68	254.74 ± 22.28	250.58 ± 35.17
VH/CD	3.05 ± 0.18^b^	4.42 ± 0.43^a^	3.56 ± 0.55^ab^	3.79 ± 0.18^ab^

VH, villus height; CD, crypt depth. 0 mg/kg (C group), 100 mg/kg (L group), 200 mg/kg (M group), and 300 mg/kg (H group) thymol were added to the basic diets of the blue foxes. The values are expressed as the means ± SD. In the same raw, means for different lowercase letters were significantly different (*P* < 0.05).

### 3.5 Changes in the gut microbiota

**FIGURE 2:**
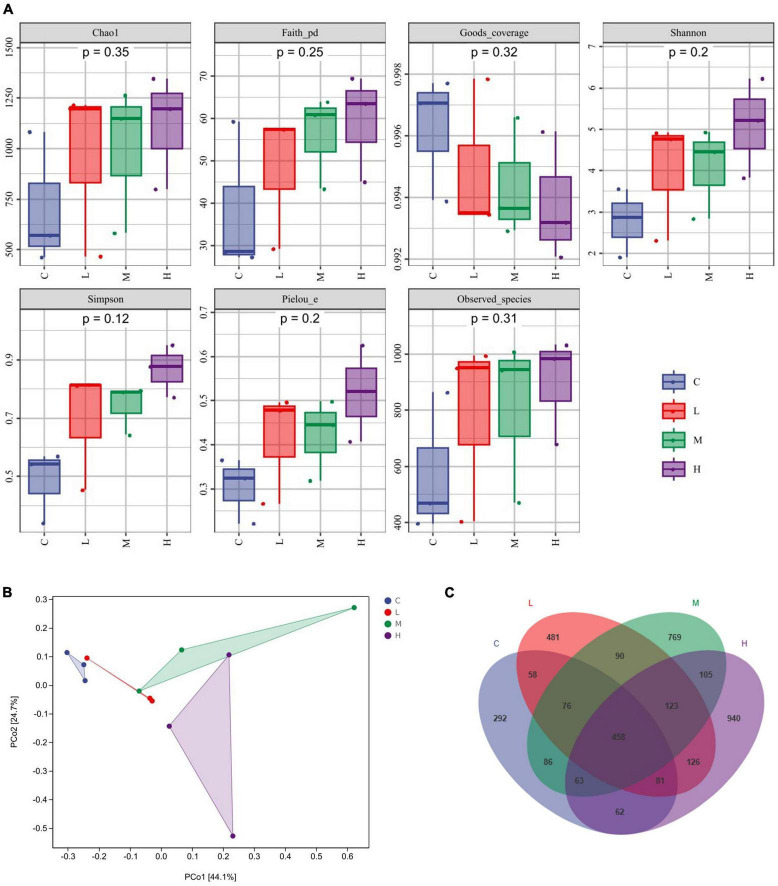
Changes in the gut microbiota. **(A)** α diversity, including Chao1, Faith, Goods, Shannon, Simpson, Pielou, and Observed indices. **(B)** PCoA analysis. **(C)** Venn diagram analysis. 0 mg/kg (C group), 100 mg/kg (L group), 200 mg/kg (M group), or 300 mg/kg (H group) thymol was added to the basic diets of the blue foxes.

α-Diversity was characterized by the Chao1 and the Observed species indices for richness, the Shannon and Simpson indices for diversity, the Faith-pd index for evolutionary diversity, the Pielou index for evenness, and the Goods coverage index for coverage. The α diversity (Figure 2A) did not significantly differ among the indices (*P* > 0.05). β-Diversity analysis was used to explore whether there were significant community differences among the samples, and the closer the distance between points was, the more similar the species composition was. As shown in Figure 2B, compared to those in the C group, the points in the other groups were further apart, indicating that thymol can alter the structure of the microbial community. Using a Venn diagram to analyze the number of unique and total OTUs in each group, we found a total of 1,176 OTUs in the C Group, 1,323 OTUs in the L Group, 1,770 OTUs in the M Group, and 1,958 OTUs in the H Group (Figure 2C). With increasing thymol addition, the number of unique OTUs increased. Among them, the C Group had 292 OTUs, the L Group had 481 OTUs, the M Group had 769 OTUs, and the H Group had 940 OTUs (Figure 2C).

### 3.7 Spearman correlation analysis

**FIGURE 3:**
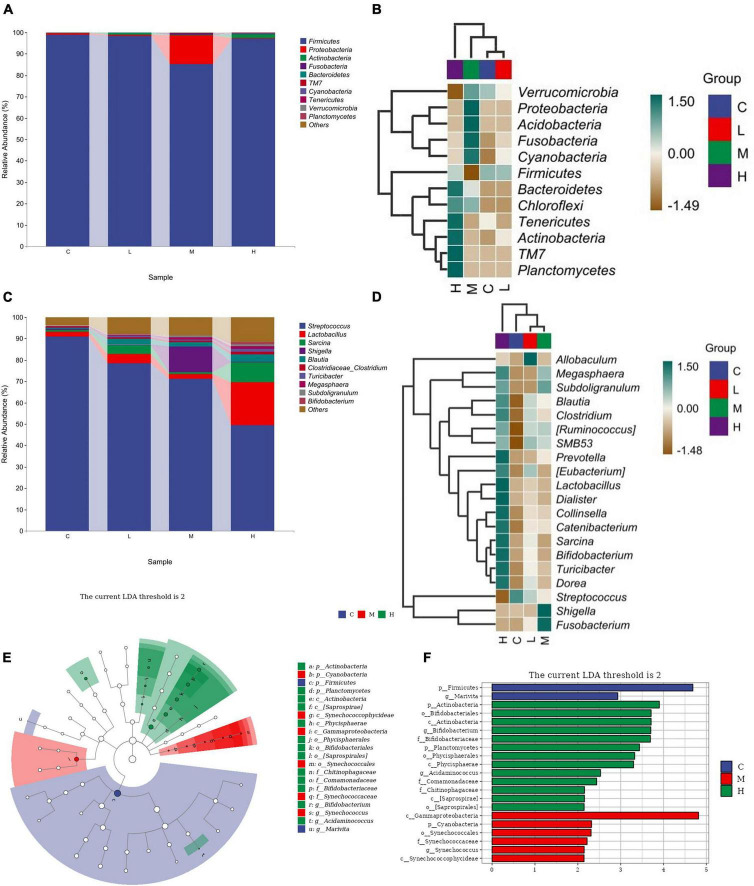
Screening of differential bacteria. **(A)** Relative abundance of bacteria at the phylum level. **(B)** Significant differences in bacteria at the phylum level. **(C)** Relative abundance of bacteria at the genus level. **(D)** Significant differences in bacteria at the genus level. **(E)** Histogram of LEfSe (log10 LDA score > 2). **(F)** Cladogram of LEfSe (log10 LDA score > 2). 0 mg/kg (C group), 100 mg/kg (L group), 200 mg/kg (M group), or 300 mg/kg (H group) thymol was added to the basic diets of the blue foxes.

The relative abundance of the gut microbiota at the phylum level is shown in Figure 3A. From the perspective of phylum-level classification, *Firmicutes*, *Proteobacteria*, *Actinobacteria*, *Fusobacteria*, and *Bacteroidetes* were the main microorganisms in the gut of blue foxes. Adding thymol to the diet can change the abundance of *Firmicutes*, *Proteobacteria*, *Actinobacteria*, *Fusobacteria*, *Bacteroidetes*, *TM7*, *Cyanobacteria*, *Tenericutes*, *Verrucomicrobia*, *Planctomycetes*, *Chloroflexi*, and *Acidobacteria* in the gut of blue foxes (Figure 3B). The dominant bacteria at the genus level were *Streptococcus*, *Lactobacillus*, *Sarcina*, *Shigella*, and *Blautia* (Figure 3C). Figure 3D shows that thymol can change the abundance of *Streptococcus*, *Lactobacillus*, *Sarcina*, *Shigella*, *Blautia*, *Clostridium*, *Turicibacter*, *Megasphaera*, *Subdoligranulum*, *Bifidobacterium*, *Collinsella*, [*Eubacterium*], *Catenibacterium*, *Fusobacterium*, *Dialister*, *Allobaculum*, [*Ruminococcus*], *Dorea*, *SMB53*, and *Prevotella*. Linear discriminant analysis effect size (LEfSe) was further performed to identify different bacterial taxa among the different groups (log10 LDA score > 2). We found that two bacterial genera, *Firmicutes* and *Marivita*, were enriched in the C group; 13 bacterial genera were particularly abundant in the H group, including *Actinobacteria*, *Bifidobacteriales*, *Actinobacteria*, *Bifidobacterium*, *Bifidobacteriaceae*, *Planctomycetes*, *Phycisphaerales*, *Phycisphaerae*, *Acidaminococcus*, *Comamonadaceae*, *Chitinophagaceae*, *[Saprospirae]*, and *[Saprospirales]* (Figures 3E, F); *Gammaproteobacteria*, *Cyanobacteria*, *Synechococcales*, *Synechococcaceae*, *Synechococcus*, and *Synechococcophycideae* were enriched in the M group; and no bacteria were enriched in the L group (Figures 3E, F).

### 3.6 Spearman correlation analysis

**FIGURE 4:**
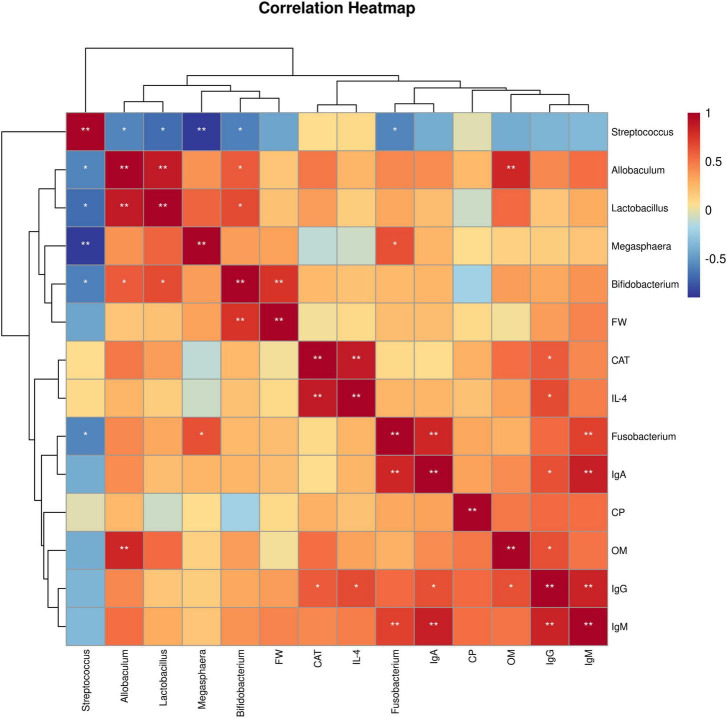
Spearman correlation analysis. Heatmap of correlations between growth performance or serum indices and the gut microbiota. FW, final weight; CAT, catalase; IL-4, interleukin-4; IgA, immunoglobulin A; CP, crude protein digestibility; OM, organic matter digestibility; IgG, immunoglobulin G; IgM, immunoglobulin M. 0 mg/kg (C group), 100 mg/kg (L group), 200 mg/kg (M group), and 300 mg/kg (H group) thymol were added to the basic diets of the blue foxes. The results of the correlation analysis were analyzed by Spearman correlation analysis; *P < 0.05; **P < 0.01.

Spearman’s rank correlation analysis was used to evaluate the correlation between production performance or serum indices and the gut microbiota. As shown in Figure 4. *Bifidobacterium* showed an extremely significant positive correlation with FW (*P* < 0.01). CAT was positively correlation with IgG (*P* < 0.05) and strongly positively correlated with IL-4 (*P* < 0.01). IL-4 was positively correlated with IgG (*P* < 0.05). *Fusobacterium* had an extremely significant positive correlation with IgM and IgA (*P* < 0.01). IgA was positively correlated with IgG (*P* < 0.05) and extremely significantly positively correlated with IgM (*P* < 0.01). OM was positively correlated with IgG (*P* < 0.05) and extremely significantly positively correlated with *Allobaculum* (*P* < 0.01). IgG had an extremely significant positive correlation with IgM (*P* < 0.01). In addition, most gut bacteria were significantly correlated with each other (*P* < 0.05).

## 4 Discussion

As a type of fur animal, the fur quality of blue foxes determines its economic benefits. The health of blue foxes is the key to determining the quality of their fur. During the breeding process, dietary supplementation with antibiotics can increase growth performance, feed intake and daily weight gain (Gomes et al., 2023). However, the excessive use of antibiotics leads to the development of antibiotic resistance in pathogenic microorganisms, which affects the economic benefits of blue foxes. Thymol, a high-quality feed additive, has the potential to replace antibiotics and has a positive effect on intestinal health and growth performance. At present, thymol has been applied in various animal production systems, but there are few reports on the impact of thymol on blue foxes. This study aimed to investigate the effects of thymol on the growth performance and intestinal health of blue foxes. The results indicated that, similar to the addition of antibiotics, the addition of thymol to the diet can improve the growth performance, apparent digestibility, and immune response of blue foxes. However, unlike antibiotics, thymol has a positive effect on promoting the colonization of beneficial bacteria in the intestine.

The primary objectives of blue fox breeding are increased efficiency and quality in fur production for clothing and accessories (Thirstrup et al., 2017). Fur quality and skin size are the most important traits in blue fox breeding and are the main determinants of sales price and subsequent income for fur producers (Thirstrup et al., 2017). Although the price of pelts largely depends on their quality, these traits can only be obtained after harvesting. Therefore, growth performance is the basis for determining the quality and yield of pelt (Moller, 1999). Our study revealed that dietary supplementation with 200 or 300 mg/kg thymol could increase FW, providing a basis for improving pelt yield. Previous studies have shown that supplementing thymol during the nursery period has a beneficial effect on the growth performance of growing pigs (Zhao et al., 2023). Adding 50 mg/kg thymol to the diet can improve the ADG of weaned piglets by promoting intestinal health (Wang et al., 2022). The beneficial effects of antibiotics on growth performance may be attributed to their antibacterial effects, but their ability to disrupt gut microbiota homeostasis cannot be ignored (Gadde et al., 2017). Our research results indicate that adding thymol to the diet can increase the FW of blue foxes without disrupting the homeostasis of the gut microbiota, providing a basis for improving pelt yield. It can be inferred that thymol can replace antibiotics to improve the growth performance and pelt yield of blue foxes.

Apparent nutrient digestibility is usually an indicator of the digestion and absorption of nutrients by animals, and it is directly related to the growth performance of blue foxes. A decrease in apparent digestibility leads to nutrient loss and affects the production profits of farmers (Liu et al., 2023). An increase in apparent digestibility can lead to an increase in the growth performance of beef cattle (Tang et al., 2023). Thymol, a healthy and green plant essential oil extract that does not cause potential side effects on the body, has been used as a food preservative, insect repellent and anti-inflammatory drug (Luo et al., 2020). In addition, the addition of 400 mg/kg essential oil containing thymol to the diet can improve the apparent digestibility of OM and CP in weaned piglets (Li Y. Y. et al., 2023). The apparent nutrient digestibility can intuitively reflect the degree of nutrient utilization by animals. The digestibility of CP and OM can affect the growth performance of blue foxes (Niu et al., 2019). Similarly, our results showed that supplementing diets with 100 mg/kg thymol can increase the apparent digestibility of OM and CP, which can increase the growth performance of blue foxes. We can infer that the increase in the apparent digestibility of OM and CP may further promote growth performance while potentially improving the pelt yield of blue foxes.

Serum indicators can serve as important indicators reflecting the growth performance of the body, as well as the physiological and health status of animals (Wang et al., 2023). The antioxidant capacity can indirectly reflect an animal’s disease resistance. A previous study has shown that adding 200 mg/kg thymol to the diet can enhance the antioxidant capacity of laying hens by increasing the serum CAT content, which is similar to our present results (Zhao et al., 2021). It can be inferred that thymol can enhance the antioxidant capacity of blue foxes. As a multifunctional immune regulatory cytokine, IL-4 is the main promoter of helper T-cell 2 (Th2) development and plays an important role in enhancing the immune system (Yang et al., 2022). The increase in the serum IL-4 concentration in our study indicated that thymol had a positive effect on the blue fox immune system. Ig is one of the main anti-infective components in the blood. It is a unique glycoprotein that protects the body from harmful bacteria, viruses, and other environmental pathogens by binding or forming encapsulation barriers (Balan et al., 2019). Previous studies have shown that Ig has multiple biological effects, including effects on growth, immunity, intestinal growth, and intestinal barrier function (Pierce et al., 2005; Maijó et al., 2012). The present study showed that adding thymol to basic diets can increase the serum IgA, IgG, and IgM levels, suggesting that thymol has the potential to improve the immune system. Similarly, it has been reported that adding thymol to the diet can significantly improve the immune response of chickens (Mosleh et al., 2013). Furthermore, the use of plant essential oils (containing thymol) in the chicken diet can enhance the phagocyte system and humoral and cellular immune responses of broilers by increasing antibody titers, playing an important positive role in resisting different infections (Pérez-Rosés et al., 2015). Thus, thymol can enhance the systemic immune response and has a positive effect on resisting the invasion of pathogenic bacteria. These results further demonstrate the potential of thymol as a substitute for antibiotics.

According to a previous report, adding thymol to the diet can not only improve the immune response but also alleviate intestinal injury in broilers by improving intestinal integrity (Du et al., 2016). The integrity of the intestinal structure is the foundation of intestinal health and normal digestion. Ensuring a healthy intestinal morphology is crucial for animal digestion of food and is crucial for improving apparent nutrient digestibility (Kogut and Arsenault, 2016). The small intestine includes the duodenum, jejunum, and ileum, and its key function is to mix acidic gastric chyme with bile to digest and absorb complex nutrients (Spiller and Hoad, 2020). Therefore, maintaining stable small intestine morphology and function is crucial for the health of the digestive system. The development of the intestine is closely related to nutrient absorption in animals, especially the VH and CD, which are important indicators for measuring intestinal digestion and absorption. An increase in VH can increase the contact area between the small intestine and intestinal contents (Houshmand et al., 2012). The VH/CD can reflect the functional status of the small intestine, and a higher VH/CD indicates that the small intestine has a greater nutrient absorption capacity and better growth performance (Wang et al., 2020). Studies have shown that the small intestine VH/CD ratio determines the protein digestibility of weaned piglets. A decrease in the VH/CD ratio leads to a decreased intestinal absorption area, which reduces protein absorption (Engelsmann et al., 2022). Our study revealed that thymol increased the VH/CD ratio. Similarly, adding 0.025% thymol to rabbit diets for 21 days can improve the VH/CD ratio of the small intestine wall, and this positive effect is still present after the withdrawal of thymol (Placha et al., 2022). Therefore, we infer that thymol may improve the apparent nutrient digestibility of blue foxes by increasing the VH/CD ratio of the small intestine.

The gut microbiota plays an important regulatory role in intestinal health, and dysbiosis of the microbiota can result in permanent alterations in the physiological response (Vamanu and Rai, 2021). Due to the low bioavailability of thymol, up to 90% of phenolic compounds enter the colon unaltered and interact with colonic bacteria (Espín et al., 2017). To investigate the positive effect of thymol on blue fox intestinal health, we detected the composition of the gut microbiota further. The stability of the gut microbiota promotes the health of the body, but the gut microbiota is influenced by various factors, such as age, disease status, and feed (Fan and Pedersen, 2021). Changes in the gut microbiota also affect animal production performance. Our study revealed that the main microbiota in the rectum of blue foxes were *Firmicutes*, *Proteobacteria*, *Actinobacteria*, *Fusobacteria*, and *Bacteroidetes*. Liu et al. (2020) also reported similar results, the main phylum-level microorganisms in the intestine of blue foxes are *Firmicutes*, *Bacteroidetes*, *Proteobacteria*, *Actinobacteria*, and *Fusobacteria*. The inconsistency in the gut microbiota structure may be attributed to differences in feed nutrition, and the addition of thymol may also be the most important factor leading to this change. A variety of factors might explain the discrepancy between studies, such as differences in diets, animals, and the environment. Treatment with dietary thymol did not notably change the proportion of the gut microbiota or bacterial diversity in laying hens or weaned pigs (Van Noten et al., 2020; Zhao et al., 2023). Similarly, our results indicate that thymol does not affect the α diversity of the gut microbiota. It can be speculated that thymol will not have a significant impact on the stability of the gut microbiota. A high-fat diet could cause an increase in *Firmicutes* and a decrease in *Bacteroidetes*, and a higher *Firmicutes*/*Bacteroidetes* ratio helps mice and piglets produce more fat (Turnbaugh et al., 2008; Li et al., 2018). According to our results, the increase in *Firmicutes* and the decrease in *Bacteroidetes* may be attributed to the increased fat content in feed by thymol, which may also be a potential factor in improving FW. The positive impact of thymol on intestinal health is its inhibitory effect on pathogenic bacteria (Placha et al., 2022). At the genus level, we found that *Streptococcus* decreased with increasing thymol content. *Streptococcus* is a gram-positive bacterial species that can cause various inflammatory diseases (Chen et al., 2023; Kuperwaser et al., 2023). The decrease in *Streptococcus* in this study may have been caused by an increase in serum Ig (Breitfelder et al., 2023). It has been reported that supplementation with *Megasphaera* improves livestock production performance, especially in terms of ADG and carcass quality (Susanto et al., 2023). Adding *Megasphaera* to sheep diets can increase feed intake and ADG by increasing the production of propionate in the rumen (Henning et al., 2010). In addition, we found that adding thymol to diets can increase the abundance of *Lactobacillus* in the intestine. As a probiotic, *Lactobacillus* has various beneficial effects, such as antimicrobial activity, immunomodulation, antioxidative effects, and antiallergenic effects (Slattery et al., 2019). Giannenas et al. (2014) found that adding 30 mg/kg thymol to the diets of male turkeys can increase the abundance of *Lactobacillus* in the cecum, thereby promoting nutrient utilization and growth performance. The strong antioxidant properties of thymol can protect intestinal epithelial cells and prevent inflammation while inhibiting pathogenic bacteria and supporting the colonization of beneficial bacteria such as *Lactobacillus* (Iqbal et al., 2020). In this study, thymol promoted the growth of blue foxes by increasing the abundance of *Megasphaera* and *Lactobacillus* and reducing the abundance of *Streptococcus*.

Through LEfSe analysis, we found that *Bifidobacterium* may be a potential differential bacterium. The genus *Bifidobacterium* belongs to the *Actinobacteria* phylum and plays key roles in the development of physiology, including the maturation of the immune system and the use of dietary components. *Bifidobacterium* has been included as a bioactive ingredient in functional foods, as well as in food supplements and drugs due to its beneficial effects (Hidalgo-Cantabrana et al., 2017). *Bifidobacterium* can regulate the immune response at the intestinal mucosal level and play a positive role in regulating the levels of Ig and inflammatory factors (Gavzy et al., 2023). In our study, the increase in the abundance of *Bifidobacterium* may have promoted the immune function of blue foxes. Furthermore, Spearman correlation analysis revealed a positive correlation between *Bifidobacterium* and FW, indicating that thymol may promote the health of blue foxes by increasing the abundance of *Bifidobacterium*. Similarly, it has been reported that supplementing with *Bifidobacterium* in the diet for 28 days can promote weight gain in Bangladeshi infants (Barratt et al., 2022). Some members of the genus *Fusobacterium* are capable of fermentative metabolism in anaerobic environments to produce organic acids, which might play a positive role in the metabolism of nutrients in the gut (Ma et al., 2022). When studying the impact of early colonization of the gut microbiota on growth performance, it was found that *Fusobacterium* could significantly increase the growth performance of lambs (Xiao et al., 2023). An increasing number of studies have shown a close relationship between *Fusobacterium* and immune regulation, and *Fusobacterium* intervenes in the occurrence of diseases by regulating the innate immune system (Zhang et al., 2023). Our study further confirmed that the increase in serum IgA and IgM may be related to *Fusobacterium*. In addition, the present study revealed that OM was positively correlated with IgG, suggesting that thymol can increase the apparent digestibility of OM by enhancing immune function (Hashemipour et al., 2013). *Allobaculum* can produce butyric acid, which plays an important role in the gastrointestinal system, particularly in regulating host fat storage. Previous studies have shown that mice fed high-fat diets or high-sucrose diets exhibit disrupted gut microbiota, particularly a decrease in *Allobaculum* abundance (Kong et al., 2019). Changes in the abundance of *Allobaculum* in the intestine were found in high-fat diet-fed mice, suggesting that *Allobaculum* may be an important microorganism regulating OM digestion ability (Zheng et al., 2021). Oxidative stress can lead to a decrease in immune function and growth performance. Our results revealed a positive correlation between CAT, immunoglobulin G (IgG), and IL, indicating that thymol, a natural antioxidant, can enhance the immune function of blue foxes, which has been validated in previous studies (Luna et al., 2017). From our study results, it can be inferred that thymol may improve the growth performance of blue foxes by increasing the abundance of *Fusobacterium*, *Bifidobacterium*, and *Allobaculum*.

At present, the problems of bacterial resistance and food-borne antibiotic residues caused by the abuse of antibiotics have aroused much concern (Bahaddad et al., 2023). With the demand for high-quality animal products, it is imperative to exploit effective and green feed additives that can stimulate latent productive capacity (Li L. X. et al., 2023). The results of the current study suggest that thymol can improve the nutritional digestion rate of blue foxes by promoting intestinal morphology, thereby enhancing their growth performance, which has a positive effect on increasing fur production. In addition, it can be inferred that thymol may improve the serum biochemical indicators of blue foxes by regulating the metabolic function of the intestinal microbiota, which can further improve their health (Singh et al., 2022). These effects were greater when the concentration of the extract was 100 mg/kg. These results suggest that thymol can replace antibiotics to improve the growth performance of blue foxes and can be further promoted for the production of other fur animals. At present, we are conducting further research on the effects of long-term thymol feeding on the growth performance and fur quality of winter fur-growing blue foxes. However, some limitations of this study should be mentioned. Due to the small number of blue foxes available for study under actual conditions, further validation of the gut microbiota results is needed. In addition, further testing of organ indicators and routine blood tests are needed to determine the effect of thymol on the growth performance of blue foxes. However, thymol is unstable and often produces unpleasant odors, which hinders its application. Microencapsulation may be an effective method to address this drawback (Mo et al., 2022). Feeding differential bacteria or conducting fecal microbiota transplantation experiments can further clarify whether thymol promotes the growth performance of blue foxes by regulating the gut microbiota. Furthermore, blood and fecal metabolomics analyses can further explore the mechanism by which thymol promotes the health of blue foxes.

## 5 Conclusion

Adding thymol to the diet can improve the body weight, apparent nutrient digestibility, antioxidant capacity, immune function, and intestinal health of blue foxes. Analysis of the gut microbiome revealed that thymol can increase FW by increasing the abundance of *Bifidobacterium*, increase the serum levels of IgA and IgM by increasing the abundance of *Fusobacterium*, and increase the apparent digestibility of OM by increasing the abundance of *Allobaculum*. The optimal amount of thymol added to the diets of blue foxes is 100 mg/kg.

## Data availability statement

The 16S rRNA gene sequencing data in the present study are available in the NCBI Sequence Read Archive (SRA) repository under accession number PRJNA1122965.

## Ethics statement

The animal studies were approved by the Experimental Animal Welfare and Ethics Committee of Jilin Agricultural University. The studies were conducted in accordance with the local legislation and institutional requirements. Written informed consent was obtained from the owners for the participation of their animals in this study.

## Author contributions

CY: Writing – original draft. SC: Methodology, Writing – original draft. RS: Investigation, Writing – original draft. LR: Investigation, Writing – original draft. TZ: Investigation, Writing – original draft. MW: Writing – review and editing. AZ: Project administration, Writing – original draft.
